# Evaluation of the Effect of an Intraperitoneal Cytostatic-Loaded Supramolecular Hydrogel on Intestinal Anastomotic Healing in an Animal Model

**DOI:** 10.3390/life13102076

**Published:** 2023-10-18

**Authors:** Danique J. I. Heuvelings, Anne G. W. E. Wintjens, Audrey C. H. M. Jongen, Maurits J. C. A. M. Gielen, Kaatje Lenaerts, Peter-Paul K. H. Fransen, Marion J. Gijbels, Geert C. van Almen, Patricia Y. W. Dankers, Ignace H. J. T. de Hingh, Nicole D. Bouvy

**Affiliations:** 1 NUTRIM School of Nutrition and Translational Research in Metabolism, Maastricht University, 6229 ER Maastricht, The Netherlands; 2 Department of General Surgery, Maastricht University Medical Center (MUMC+), 6202 AZ Maastricht, The Netherlands; 3 Department of General Surgery, Catharina Ziekenhuis, 5623 EJ Eindhoven, The Netherlands; 4 GROW-School for Oncology and Reproduction, Maastricht University, 6229 ER Maastricht, The Netherlands; 5 UPyTher BV, 5612 AE Eindhoven, The Netherlands; 6 Department of Pathology, Maastricht University Medical Centre, 6202 AZ Maastricht, The Netherlands; 7 Department of Medical Biochemistry, Experimental Vascular Biology, Amsterdam Infection and Immunity, Amsterdam Cardiovascular Sciences, Amsterdam University Medical Center, 1105 AZ Amsterdam, The Netherlands; 8 Institute for Complex Molecular Systems, Eindhoven University of Technology, 5612 AZ Eindhoven, The Netherlands; 9 Department of Biomedical Engineering, Laboratory of Chemical Biology, Eindhoven University of Technology, 5612 AZ Eindhoven, The Netherlands; 10 Department of Chemical Engineering & Chemistry, Eindhoven University of Technology, 5612 AZ Eindhoven, The Netherlands

**Keywords:** peritoneal metastases, colorectal cancer, intraperitoneal delivery, injectable supramolecular hydrogel, mitomycin C

## Abstract

The prognosis of colorectal cancer patients with peritoneal metastases is very poor. Intraperitoneal drug delivery systems, like supramolecular hydrogels, are being developed to improve local delivery and intraperitoneal residence time of a cytostatic such as mitomycin C (MMC). In this study, we evaluate the effect of intraperitoneal hydrogel administration on anastomotic healing. Forty-two healthy Wistar rats received a colonic end-to-end anastomosis, after which 6 animals received an intraperitoneal injection with saline, 18 with unloaded hydrogel and 18 with MMC-loaded hydrogel. After 7 days, animals were euthanized, and the anastomotic adhesion and leakage score were measured as primary outcome. Secondary outcomes were bursting pressure, histological anastomosis evaluation and body weight changes. Twenty-two rats completed the follow-up period (saline: *n* = 6, unloaded hydrogel: *n* = 10, MMC-loaded hydrogel: *n* = 6) and were included in the analysis. A trend towards significance was found for anastomotic leakage score between the rats receiving saline and unloaded hydrogel after multiple-comparison correction (*p =* 0.020, α = 0.0167). No significant differences were found for all other outcomes. The main reason for drop-out in this study was intestinal blood loss. Although the preliminary results suggest that MMC-loaded or unloaded hydrogel does not influence anastomotic healing, the intestinal blood loss observed in a considerable number of animals receiving unloaded and MMC-loaded hydrogel implies that the injection of the hydrogel under the studied conditions is not safe in the current rodent model and warrants further optimalisation of the hydrogel.

## 1. Introduction

Colorectal cancer (CRC) is the third most prevalent cancer type worldwide and a common cause of morbidity and mortality generally attributable to metastatic disease [1,2]. The prognosis of CRC patients with peritoneal metastases (PM) is very poor. For a selective group of patients, there are life-prolonging treatment options available. A common strategy for physically fit patients with limited disease burden is cytoreductive surgery (CRS) with or without adjuvant hyperthermic intraperitoneal chemotherapy (HIPEC) [1,3]. Patients who are not considered eligible may undergo a new palliative treatment option that is currently being investigated called pressurized intraperitoneal aerosol chemotherapy (PIPAC) [4,5,6,7,8].

Despite the introduction of HIPEC and PIPAC, treatment failure is still a major issue in CRC patients with PM. As intraperitoneal delivery of cytostatic drugs is the preferred route for PM treatment, intraperitoneal drug delivery systems are being investigated [9]. One such system is a supramolecular hydrogel, which has shown potential in the field of PM therapy. The development of targeted therapies using drug-loaded hydrogels can help deliver drugs directly to the affected area, improving therapeutic outcomes [10,11]. In recent years, our research team has conducted several experiments investigating the feasibility, safety, tissue compatibility and therapeutic efficacy of a supramolecular hydrogel loaded with mitomycin C (MMC) [12,13]. The main function of this injectable hydrogel is to form an intraperitoneal depot of slow-releasing MMC, aiming to establish prolonged exposure of the PM to the cytostatic agent. The therapeutic efficacy in a PM rat model was investigated before and demonstrated that there is a clinically relevant survival benefit for MMC-loaded hydrogel compared to injection of free MMC [13]. 

Intraperitoneal chemotherapy is usually preceded by cytoreductive surgery, which frequently includes a colon resection to remove the primary tumor, often requiring a colonic or colorectal anastomosis. Anastomotic leakage (AL) is considered one of the most important complications after such a colon resection. It occurs in 1 to 19% of the cases and has a negative impact on survival [14,15]. Chemotherapeutics, including MMC, that are administered intraperitoneally are suspected to have an effect on anastomotic healing after surgery [16,17]. As the therapeutic benefit of MMC-loaded hydrogel in PM has been demonstrated by previous work, it is crucial to investigate its influence on anastomotic healing before considering this treatment option for in combination with CRS for PM of colorectal origin in humans. 

The aim of this study was to investigate whether intraperitoneal administration of hydrogel (both unloaded and MMC-loaded) affects colonic anastomotic healing; specifically, whether it results in a higher incidence of AL in a rodent animal model. For this purpose, the previously investigated supramolecular hydrogel was intraperitoneally applied in healthy rats after creating a sufficient end-to-end colon anastomosis.

## 2. Methods

### 2.1. Ethics and Safety Protocol

This animal study was performed at the animal center of Maastricht University (Maastricht, The Netherlands). The experimental protocol followed the Dutch Animal Experimental Act and was approved by the Animal Experimental Committee of Maastricht University Medical Center (project license AVD1070020198765). The ARRIVE guidelines [18] for reporting animal research were followed and additional information can be found in the Supplementary Materials. During the experiment, we maintained a local cytostatic protocol developed by the animal center of Maastricht University to ensure appropriate safety measures while working with chemotherapy.

### 2.2. Animals and Housing

A total of 42 healthy adult Wistar rats (21 males/21 females) aged 10–12 weeks with a body weight of 400 g–500 g (males) and 230 g–330 g (females) were used (all characteristics can be found in Supplementary Table S1). All rats were bred by Charles River Laboratories (Sulzfeld, Germany). An acclimatization period of at least one week was maintained. All animals were socially housed in individually ventilated cages in a temperature- and humidity-controlled room with 12 h light/dark cycles. All animals had ad libitum access to food (10 mm Sniff rat/mouse sterilized food compressed into pallets) and acidified drinking water. Postoperatively, animals were weighed daily, and welfare was scored systematically based on predefined standardized welfare scoring sheets (Supplementary Materials). Human endpoints (HEPs) were defined prior to the experiment. 

### 2.3. Study Design, Randomization and Blinding

The aim of this study was to investigate if intraperitoneal administration of MMC-loaded and unloaded hydrogel affected the anastomotic healing compared to animals receiving a peritoneal injection with saline. To study the effect on anastomotic healing, all animals received a sufficient colon–colon anastomosis. Subsequently, the animals were randomly assigned to one of the three following intervention groups receiving a single injection with either saline (*n* = 6), unloaded hydrogel (*n* = 18) or MMC-loaded hydrogel (*n* = 18). The random allocation of the animals was performed by a computer-based random order generator. After a follow-up period of seven days (most ALs show up within the first week after surgery), the anastomotic healing was assessed. During the allocation, the conduct of the experiment and the outcome assessment, the research team, the veterinarian and the people working in the animal facility were blinded for the group allocation. 

### 2.4. Supramolecular Hydrogel

For this experiment, we used a supramolecular hydrogel based on polyethylene glycol (PEG) chains end-modified with fourfold hydrogen bonding the ureido-pyrimidinone (UPy) units (UPy–PEG Hydrogel), previously described by Wintjens et al. [12,13,19,20]. Identical to the previous studies described by Wintjens et al., the rats received 20 mL/kg of hydrogel corresponding to a single intraperitoneal injection of 5 mL for female rats (±250 g) or 8 mL for male rats (±400 g).

### 2.5. Anesthesia, Surgical Procedure and Analgesia

A subcutaneous injection of 0.05 mg/kg buprenorphine (Richter Pharma AG, Wels, Austria) was given one hour prior to surgery as an analgesic. The surgical procedure was performed by experts (A.J. and N.B.) certified for performing anastomotic models in laboratory animals. All animals underwent general anesthesia using 4–5 vol.% isoflurane supplied with air (IsoFlo, Zoeties B.V., Rotterdam, The Netherlands) for induction which was maintained with 2–3 vol.%. The body temperature was maintained by placing the animals on a heated plate with a temperature of ca. 36 °C. A 5 cm craniocaudal midline incision of the skin and abdominal musculature was performed with a scalpel, after removing the abdominal fur with electric clippers and local injection of bupivacaine (Aurobindo Pharma BV, Baarn, The Netherlands). The cecum and additional intestines were taken outside the abdomen onto sterile gauzes hydrated with sterile saline solution to prevent dehydration. The site for colon–colon anastomosis was identified at ca. 4 cm ab ani, whereafter the colon was fully transected with scissors. An end-to-end anastomosis was created using at least 9 interrupted polypropylene sutures (Prolene 6-0, Ethicon, Johnson & Johnson; Supplementary Figure S1). After the creation of a sufficient anastomosis, it was tested for leakage of water by injection of NaCl via the rectum. In case of water leaking through the anastomosis, additional sutures were placed until the anastomosis remained dry. Thereafter, the intestines were repositioned in the abdomen, and the abdomen was closed with a running suture for the muscle layer (Prolene 4-0, Ethicon, Inc., Johnson & Johnson, Raritan, NJ, USA) and interrupted sutures for the skin (Monocryl 4-0, Ethicon, Inc., Johnson & Johnson). Subsequently, the animals received a single intraperitoneal injection with 5 or 8 mL (F/M) saline, unloaded hydrogel or MMC-loaded hydrogel, corresponding to a volume-to-weight ratio of 20 mL/kg. 

Postoperatively, a saline + 3% glucose solution (3–5 mL) was administered subcutaneously to prevent dehydration. General anesthesia using isoflurane was maintained for at least 20 min after intraperitoneal administration of either one of the three interventions, conforming previous experiments with a comparable hydrogel formulation [12]. Subcutaneous injections of 0.03 mg/kg buprenorphine were continued every six hours for 48 h for all animals, as most post-operative discomfort was expected in the first 48 h. In addition, 200 mg/kg paracetamol (Dafalgan, UPSA, France) was given in a separate drinking bottle during the entire experiment. If animals showed signs of discomfort based on the welfare scoring sheets, additional pain medication by subcutaneous injections of buprenorphine was administered and/or saline + 3% glucose solution in case of dehydration signs. After seven days, all animals were euthanatized via CO_2_ asphyxiation. Afterwards, the intraabdominal cavity was inspected via laparotomy. If needed, blood samples were taken from the vena cava. 

### 2.6. Study Outcomes

The primary study outcome was macroscopic scores including anastomotic adhesion and leakage scores. Secondary outcomes were bursting pressure, histological evaluation of the anastomosis (inflammation, fibroblast activity, neoangiogenesis and oedema) and changes in body weight. 

#### 2.6.1. Macroscopic Evaluation 

After euthanasia of the animal, adhesions to the anastomotic site were assessed according to the method described by van der Ham et al. [21], in which the following classification was used: (0) no adhesions; (1) minimal adhesions, mainly between the anastomosis and omentum; (2) moderate adhesions, i.e., between omentum and the anastomotic site and between the anastomosis and a loop of the small bowel; and (3) severe and extensive adhesions, including abscess formation. The adhesion score was calculated per group by summing the scores per animal. Subsequently, AL was scored using a four-score system [22]. The latter is categorized as (1) no AL, (2) small abscess < 1 cm^3^ at the anastomotic site, (3) large abscess of >1 cm^3^ at the anastomotic site and (4) complete dehiscence with (fecal) peritonitis.

#### 2.6.2. Bursting Pressure 

Anastomotic strength was assessed by measuring the bursting pressure (Supplementary Figure S2), based on previously described methods [22,23]. In short, a 4 cm segment of the colon including the anastomosis was resected en bloc, without removal of adherend adhesions to prevent iatrogenic damage. A plastic tube was inserted in the proximal end and ligated with polypropylene 6-0 suture (Prolene 6-0, Ethicon, Inc., Johnson & Johnson). The part distal of the anastomosis was clamped. The resected colon segment was immersed in water, while air was infused using a balloon connected to a manometer (Digitron, part of Rototherm Group). The pressure (mBar) was manually increased by pumping up the balloon and inflating the colon. Bursting pressure was defined as the intraluminal pressure at which air leakage was initially observed from the anastomosis. 

#### 2.6.3. Tissue Preparation and Histological Evaluation 

After measuring the bursting pressure, the colon tissue including the anastomosis was placed in formalin. All samples were paraffinized within one week. From each formalin-fixed, paraffin-embedded (FFPE) tissue specimen, a 5 μm section was cut and stained with standard hematoxylin-eosin (H&E). Infiltration of inflammatory cells, fibroblast activity, oedema and neoangiogenesis at the anastomotic site were assessed by an experienced animal pathologist (MG). Inflammatory parameters were scored based on the modified 0-to-4 Ehrlich and Hunt numerical scale: 0  =  no evidence, 1  =  occasional evidence, 2  =  light scattering, 3  =  abundant evidence and 4  =  confluent cells or fibers [24,25]. All other characteristics were score on a 0-to-3 scale, meaning 0 = no evidence, 0.5 = minimal, 1 = mild, 2 = moderate and 3 = severe. Additionally, all other abdominal organs were collected as well and placed in formalin in case of need for further histological examination.

### 2.7. Statistical Analysis

General characteristics of the animals can be found in Supplementary Table S1. Statistical analysis was performed using SPSS (IBM SPSS Statistics for Apple, Version 27, Armonk, New York, NY, USA) and GraphPad Prism (GraphPad software for Apple, version 8.0.0, San Diego, CA, USA). Numerical variables were presented as median with interquartile range (IQR, Q1–Q3). To evaluate the statistical significance of numerical variables differences observed between groups, non-parametric tests (overall Kruskal–Wallis and post hoc Mann–Whitey U-tests for pairwise comparison) were applied. In case of significant overall tests (α = 0.05), a Bonferroni correction was used for the pairwise comparisons (α = 0.0167). The percentage of body weight change was calculated by subtracting the daily measured weight from the baseline weight of each animal. Group comparison of mean body weight was performed with mixed-effect models. 

## 3. Results

A total of 42 healthy rats underwent the surgical procedure (saline *n* = 6, unloaded hydrogel *n* = 18 and MMC-loaded hydrogel *n* = 18) of which 22 completed the follow-up period of 7 days (Supplementary Figure S3, saline = 6, unloaded hydrogel = 10, MMC-loaded hydrogel = 6) and were included in the final analysis.

### 3.1. Anastomotic Adhesion and Leakage Scores 

**Figure 1:**
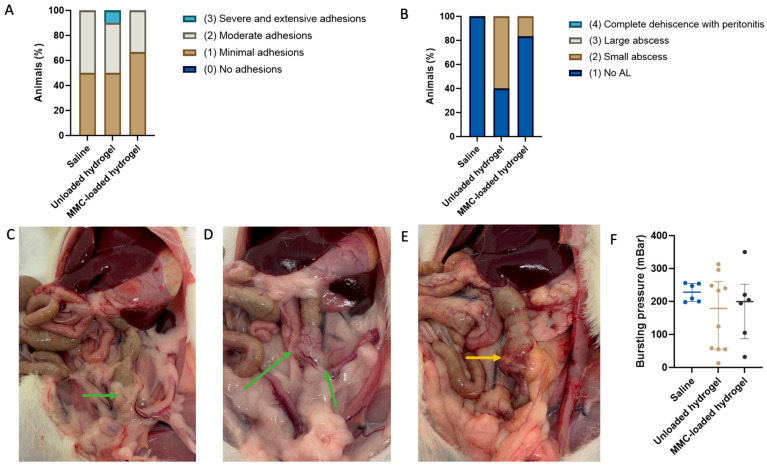
(**A**) Macroscopic anastomotic adhesion score. (**B**) Mascroscopic AL scores per group. (**C**) Minimal adhesions (green arrow) in a female rat treated with MMC-loaded hydrogel. (**D**) Moderate adhesions (green arrows) in a female saline-treated rat. (**E**) Small abscess (orange arrow) in a male rat treated with unloaded hydrogel. (**F**) Bursting pressures per group. Medians are indicated and whiskers show the Q1–Q3.

The macroscopic anastomotic adhesion and leakage scores are displayed per intervention group in Figure 1A,B. Representative images of the anastomoses with corresponding scores are shown in Figure 1C–E. The median (IQR) adhesion scores were 1.5 (1–2), 1.5 (1–2) and 1 (1–2) for saline, unloaded hydrogel and MMC-loaded hydrogel groups, respectively. There were no significant differences between the groups (Table 1). Severe and extensive adhesions were only present in one animal that had unloaded hydrogel administered. The median (IQR) AL scores were 1 (1–1), 2 (1–2) and 1 (1–1.25) for saline, unloaded hydrogel and MMC-loaded hydrogel groups, respectively. A difference was observed for the AL score (*p* = 0.034), for which pairwise comparison showed a difference for AL score comparing the saline and unloaded hydrogel subgroup (*p =* 0.020, Figure 1B). This difference was not significant after Bonferroni correction (α = 0.0167). 

### 3.2. Bursting Pressure 

The median bursting pressure was 228 (200–255) mBar, 179 (55–260) mBar and 200 (86–253) mBar for saline, unloaded hydrogel and MMC-loaded hydrogel group, respectively. This was not significant different between the groups (Figure 1F). 

### 3.3. Microscopic Evaluation

**Figure 2:**
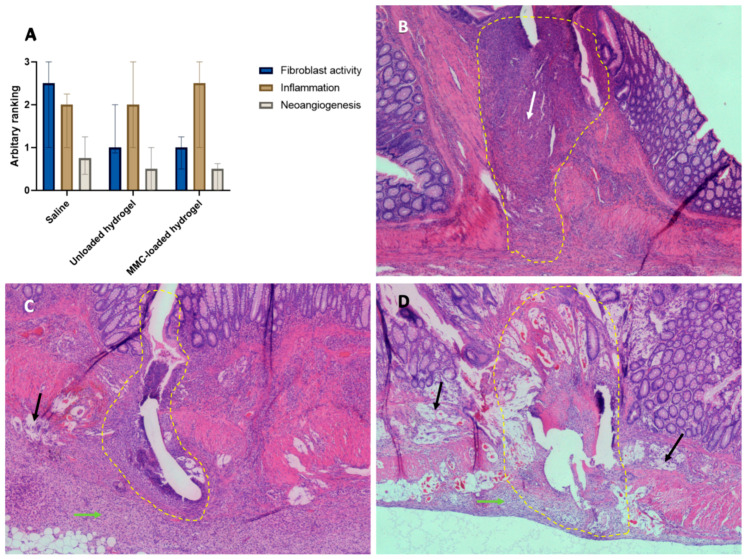
(**A**) Histological analysis of animals at postoperative day 7: scores of fibroblast activity, inflammation and neoangiogenesis. Medians are indicated and whiskers show the Q1–Q3. (**B**) Hematoxylin & Eosin (H&E) staining of anastomotic site (10 × 5) of a saline-treated animal. (**C**) H&E staining of anastomotic site (10 × 5) of an animal that received unloaded hydrogel. (**D**) H&E staining of anastomotic site (10 × 5) of animal that received MMC-loaded hydrogel. Yellow dashed line indicates site of anastomosis, white arrow highlights fibroblast activity, black arrows show the lymphangiectasia and green arrows point the area with vacuolated macrophages.

The anastomotic site of the rats which completed the experiment was microscopically scored by an experienced animal pathologist. No significant differences were found for fibroblast activity, inflammation and neoangiogenesis scores at the anastomotic site (Figure 2A). In all animals of the experimental groups (treated with MMC-loaded and unloaded hydrogel), we observed lymphangiectasia, oedema in the muscularis propria and vacuolated macrophages around the anastomotic site and in the surrounding peritoneal fat that contained foreign material (Figure 2C,D showing representative images). These histological observations were not seen in the control animals receiving normal saline (Figure 2B). 

### 3.4. Drop-Out

A considerable number of rats, 20 out of 42, were prematurely excluded from the experiment. Twelve rats were prematurely sacrificed due to visible anal blood loss (*n* = 5 unloaded hydrogel, *n* = 7 MMC-loaded hydrogel). Five additional rats (*n* = 1 unloaded hydrogel, and *n* = 4 MMC-loaded hydrogel) were taken out because of a too high welfare score and reaching HEP before the end of the experiment. One female rat treated with unloaded hydrogel was excluded for further analysis due to technical error during the operation (too much blood loss during the creation of the anastomosis). Another female unloaded hydrogel animal was taken out of the experiment after one day because the animal had opened its fascia and peritoneum, and another one treated with MMC-loaded hydrogel because she had an incarceration of omentum in an abdominal hernia.

**Figure 3:**
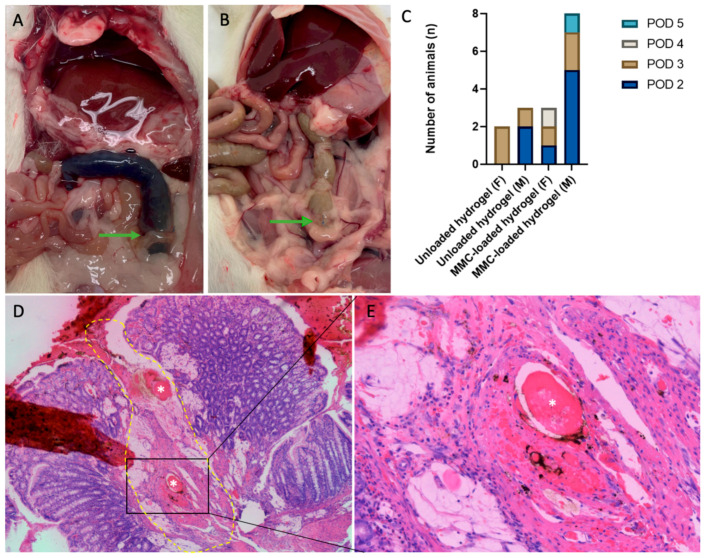
Anastomotic site (green arrows) in (**A**) an MMC-loaded hydrogel-treated rat with blood loss on POD 3 and (**B**) an MMC-loaded hydrogel-treated rat without blood loss on POD 7. Both show fat adhesions. (**C**) Occurrence of blood loss in relation to PODs. (**D**) H&E staining of anastomotic site (10 × 5) of an MMC-loaded hydrogel treated animal with intraluminal blood loss. A yellow dashed line indicates the site of anastomosis. Thrombi are annotated by asterisks, with surrounding signs of bleeding. (**E**) Enlargement (20 × 5) of the region of interest shown in (**D**), with hemorrhagic spots. M = male, F = female.

After obduction of the prematurely sacrificed rats, 16/20 rats (*n* = 5 unloaded hydrogel, *n* = 11 MMC-loaded hydrogel; 69% male) had signs of intraluminal blood loss at the anastomotic site (Figure 3A). It seemed like the blood clots accumulated at the site of the anastomosis, which was not seen in animals without blood loss (Figure 3B), and sometimes blood clots in the colon or small intestine proximal of the anastomosis were observed. We did not observe hemoperitoneum in any of the rats, nor did we identify any intraluminal blood loss in the animals surviving the whole experiment. In 50% (*n* = 8) of the animals that had intraluminal blood loss, the blood loss was present on postoperative day (POD) 2, and in 33% on POD 3; two other rats were diagnosed with blood loss on POD4 and 5 (Figure 3C). In 50% (*n* = 8), microscopic signs of blood loss around the anastomotic site were seen, e.g., necrotic blood vessels (Figure 3D,E), hemorrhagic spots in different layers (serosa, muscularis mucosa and the mucosa) or congestion in some villi. The small intestine and stomach did not show any signs to which the blood loss could be related. 

Blood samples were taken from animals with (8 rats treated with MMC-loaded hydrogel) and without (8 rats treated with unloaded hydrogel and 3 with MMC-loaded hydrogel) intestinal blood loss (Supplementary S2, Figure S4 and Table S2). Thrombocyte numbers were not different between animals with and without blood loss. In addition, coagulation factors (prothrombin time, international normalized ratio, and activated partial thromboplastin time) were estimated in 5 of the previous animals (all MMC-loaded hydrogel) of which 4 were presenting with blood loss and one did not. No abnormalities related to coagulation outcomes were found. 

### 3.5. Weight Loss and Welfare Scores

The results of the daily body weight monitoring in all animals who successfully underwent the operation are shown in Supplementary Figure S5. All animals had an initial weight gain on day 1 related to hydrogel and saline administration, followed by weight loss. From day 3, recovery to mean baseline weight was observed in saline treated animals, while animals who had hydrogel administered (unloaded hydrogel or MMC-loaded hydrogel) kept on losing weight. In both hydrogel groups, the course of the body weight was comparable, although we observed a little higher weight loss in the MMC-loaded hydrogel treated animals. A mixed-effects model showed a significant difference for both female and male rats (*p <* 0.0001 for both sexes) in favor of the saline-treated group.

Animals in both the unloaded hydrogel and MMC-loaded hydrogel had higher post-operative welfare scores, implicating more discomfort compared to animals treated with saline. Seven rats opened their laparotomy wound (*n* = 5 unloaded hydrogel, *n* = 1 MMC-loaded hydrogel, *n* = 1 saline).

## 4. Discussion

In this experiment, 42 healthy rats underwent a laparotomy to create a sufficient colonic end-to-end anastomosis to investigate whether a single intraperitoneal administration of a (drug-loaded) hydrogel affects anastomotic healing compared to saline administration. Twenty-two animals who completed the follow-up period of the experiment were included in the analyses, investigating macroscopic and microscopic anastomotic healing. Adhesion scores were not significantly different between groups. A higher AL score was found in the animals treated with unloaded hydrogel as compared to saline-treated animals, which did not remain significant after correction for multiple testing. A wider range of bursting pressure values was found in the hydrogel-treated groups compared to the saline-treated group, but the differences were not significant. In addition, fibroblast activity, inflammation and neoangiogenesis scores were not different between groups. Unexpectedly, intraperitoneal administration of unloaded and MMC-loaded hydrogel after anastomotic surgery did not prove safe due to intestinal blood loss in nearly half of the hydrogel-treated animals under the current study conditions.

Animals that received unloaded hydrogel administered had a higher, but not significant, median AL score compared to animals treated with saline. The observed difference was attributed to the occurrence of small abscesses in several of the unloaded hydrogel-treated animals. However, no large abscesses or complete dehiscence with peritonitis were found in these animals. The hydrogel-treated animals demonstrated a wide range of bursting pressure values compared to the saline-treated animals. Previous studies reported wide ranges of bursting pressure values on different PODs [26,27]. Bosmans et al. published a mean bursting pressure of 104.1 ± 40.8 mBar on POD 7 in their control group. In contrast, Kosmidis et al. reported a higher mean bursting pressure of 198.38 ± 12.80 mBar and de Castro Durães et al. even of 267.07 mBar in control animals on POD 7 [27,28]. In our cohort, 18/22, 16/22 and 3/22 animals had a bursting pressure above 104, 198 and 267 mBar, respectively. Although our measured bursting pressures seem to be in line with previously reported absolute values, it is noteworthy that the range in both hydrogel-treated groups is wider compared to the saline-treated group. In a few animals, we measured rather low bursting pressures, which may indicate disturbed anastomotic healing. Still, no large abscesses or complete dehiscence with fecal peritonitis were found. Although using the bursting pressure is the most reliable method of mechanical power assessment of the anastomosis [29], the wide range of values in the literature and our study may suggest this method is not optimal for AL assessment. 

Importantly, as this study involves an anastomotic safety model (in which normal healing is expected), we did not have to sacrifice an animal before the end of the experiment due to defective anastomotic healing or AL, nor did we identify animals with large abscesses or peritonitis. Already back in 1991, Fumagilli et al. investigated the effects of intraperitoneal chemotherapy on jejunal anastomotic healing in rats [16]. Although different types of rats, location of the anastomosis and dose of MMC, histological examination of the anastomoses in rats given an intraperitoneal bolus of 2 mg/kg MMC showed significantly slower anastomotic healing, with an incidence of AL of 52.8% after 7 days. An investigated explanation for this impaired anastomotic healing is the affected collagen synthesis, as this is an essential feature of anastomotic healing in the intestine [16]. The strength of anastomosis is influenced by the interplay between newly-synthesized and deposited collagen, as well as the degradation of preformed collagen [17]. In the initial post-operative phase (3–5 days after surgery), there is a notable decrease of up to 40% in collagen concentration near the anastomosis site, primarily attributed to increased collagenase activity at the anastomosis site [16,17]. However, starting from day 5 onwards, there is a gradual rise in collagen synthesis, leading to a progressive increase in the strength of the anastomosis. By the 7th day after surgery, the anastomosis achieves approximately 50% of its measured strength [16,30]. MMC halts the proliferation of fibroblasts, which play vital roles in several crucial aspects of the previous wound-healing process [17]. Previous experiments showed that intraperitoneal MMC administered on or after the 5th day after anastomosis creation had no significant effect on the anastomotic healing anymore [17]. As the injectable hydrogel used in our study forms an intraperitoneal depot of slow-releasing MMC, we hypothesized less impaired anastomotic healing due to the slow-releasing characteristics. This was confirmed by the finding that we did not observe any rats suffering from AL as reported in other studies [16,17]. No significant differences could be observed between the two experimental groups (unloaded hydrogel vs. MMC-loaded hydrogel) suggesting that slow-releasing but prolonged exposure of the chemotherapeutic does not impair wound healing, nor does the hydrogel. However, our results do show, although not significant, reduced fibroblast activity in unloaded hydrogel and MMC-loaded hydrogel treated animals. Despite the reduction in AL incidence in our study, we did not gain a clinical improvement due to the drop-out of almost half of the animals. 

Active hemorrhage was only seen intraluminal at the site of the anastomosis and not in the abdominal cavity. Our previous study, in which this hydrogel was applied in a rat PM model, did not demonstrate intraluminal, extensive blood loss. The main reasons for intraluminal blood loss in animal experiments are (1) a *Clostridium piliforme* or *Clostridium perfrigens entertoxin* infection, (2) intestinal ulcer formation and (3) a systemic coagulation problem. In rats suffering from intestinal hemorrhage in the current experiment, these potential causes were all excluded by follow-up analysis of feces, tissue and blood samples. After ruling out several probable causes of blood loss in animal experiments, we propose an explanation based on microscopic findings. We observed lymphangiectasia, edema in the muscularis propria and vacuolated macrophages around the anastomotic site and in the surrounding peritoneal fat that contained foreign material, also reported by Wintjens et al. [12]. We hypothesize that the hydrogel is partly absorbed by the intestinal lymph system and macrophages, causing local congestion, which causes blood vessel damage around the anastomosis. The degree of damage ranged from larger necrotic blood vessels to hemorrhagic spots in different layers (serosa, muscularis mucosa and the mucosa). Although we did not see these microscopic signs of damage in all sections of animals with intestinal blood loss, we did see large lymph congestion in all hydrogel-treated animals. Additionally, the presence of a considerable volume of hydrogel in the peritoneal space may lead to a high intraabdominal pressure which contributes to this local thrust in the first hours after injection. Based on this hypothesis, we can assume that of the animals that were taken out of the experiment due to the blood loss, the anastomotic healing was influenced due to the impaired blood supply as they were not included in the data analysis. 

An important observation was the discomfort of the animals after the surgery. Our research team and the animal facility have ample experience with rat anastomosis research [22,31] and with the intraperitoneal administration of the used hydrogel formulation [12,13]. During this experiment, five rats treated with unloaded hydrogel, one treated with MMC-loaded hydrogel and one saline-treated animal managed to open their laparotomy wound resulting all in re-interventions and the sacrifice of one of the animals, suggesting abdominal discomfort. Therefore, in all subsequently operated animals, the skin was closed with metal clips in addition to the sutures. In addition to this discomfort, control animals stabilized their weight from 3 days postoperatively, while hydrogel-treated animals kept on losing weight and almost all reached a HEP on day 7 based on the weight loss. The drop-out, general discomfort and decreased body weight were more prominent in hydrogel-treated animals, and more specific in the male animals compared to female animals, which was also reported in previous experiments [12]. 

This is the first experiment to investigate the anastomotic safety of intraperitoneal administration of unloaded and MMC-loaded hydrogel (UPy–PEG). Outcomes of interest were compared with control animals receiving saline and undergoing identical study procedures and follow-up time. This study has some limitations. Due to the high number of dropouts, the sample size of animals that completed the experiment is small and lower than predefined in the power calculation. Given the small sample size, the study results should be interpreted with caution. Paradoxical to the observation that the MMC-loaded and unloaded hydrogel is not significantly causing more AL, we report unexpected signs of extensive intestinal blood loss in almost half of these animals in this model. As the cause of the intestinal blood loss at the anastomotic site after hydrogel injection is still hypothetical, further research to reveal this observation provides insight in anastomotic healing.

## 5. Conclusions

In this rodent model, we demonstrated the influence of an intraperitoneal injectable cytostatic (MMC) loaded hydrogel (UPy–PEG) on colon anastomoses. Although our preliminary results suggest that intraperitoneal administration of the hydrogel with or without MMC does not affect anastomotic healing based on the anastomotic adhesion and leakage score, bursting pressure and microscopic evaluation, we must conclude that injection of both unloaded and MMC-loaded hydrogel under the studied conditions is not safe in the current rodent model for colorectal anastomotic surgery because of the high number of rats which were prematurely sacrificed due to intestinal blood loss. This warrants future experiments to optimize the hydrogel for use in combination with colorectal surgery.

## Figures and Tables

**Table 1:** Macroscopic adhesion and AL scores.

	Saline (*n* = 6)	Unloaded Hydrogel (*n* = 10)	MMC-Loaded Hydrogel (*n* = 6)	*p* Value
**Adhesion score** *–* ***median (Q1*** *–* ***Q3)***	1.5 (1–2)	1.5 (1–2)	1 (1–2)	0.741 ^a^
**AL score** *–* ***median (Q1*** *–* ***Q3)***	1 (1–1)	2 (1–2)	1 (1–1.25)	**0.034**^a,^*

^a^ Kruskal–Wallis test; * *p =* 0.020 after pairwise comparison of saline and unloaded hydrogel group with Mann–Whitney U test, which was not significant after Bonferroni correction.

## Data Availability

Data supporting this study are included within supporting materials. More information can be gained through contacting the corresponding author.

## Supplementary Materials

The following supporting information can be downloaded at: https://www.mdpi.com/article/10.3390/life13102076/s1. S1: Additional supplementary information according to ARRIVE guidelines and S2: Supporting materials (results); Table S1. Rat characteristics and outcomes, Table S2. Individual results of additional coagulation tests in four rats with (+) and one without (−) intestinal blood loss., Figure S1. Surgical procedure: creation of the anastomosis + closure of the fascia., Figure S2. Bursting pressure measurement., Figure S3. Survival proportions of the whole cohort., Figure S4. Thrombocyte values of animals with (+) and without (−) blood loss. Figure S5. Median body weight changes in percentages compared to mean baseline weight in the 7 days of the experiment. Refs. [32,33,34,35] have been cited in supplementary materials.
