# Supplementary material for: Evaluation of the Effect of an Intraperitoneal Cytostatic-Loaded Supramolecular Hydrogel on Intestinal Anastomotic Healing in an Animal Model
Source: Life (Basel). 2023 Oct 18;13(10):2076. doi: 10.3390/life13102076 (PMC10608244; doi:10.3390/life13102076)
Supplement: Supplementary file 1 [file life-13-02076-s001.zip › life-2593339-supplementary.pdf]

S1: Additional supplementary information according to ARRIVE guidelines

Ethical statement

This animal study was performed at the animal center of Maastricht University (Maastricht, The Netherlands). The experimental protocol followed the Dutch Animal Experimental Act and was approved by the Animal Experimental Committee of Maastricht University Medical Center (project license AVD1070020198765).

Study design and sample size

For this experiment 42 healthy adult male and female Wistar rats were used. The research question of this experiment was whether the incidence of anastomotic leakage (AL) in animals treated with unloaded or MMC-loaded hydrogel is the same as in animals without any treatment. The latter was investigated using a rat model with a sufficient colon-colon anastomosis. The unloaded or MMC-loaded hydrogel treated animals were compared with animals subjected to saline. The rule of three from Viechtbauer was used for the sample size calculation [1]. This power calculation aims at a 95% (γ) chance of a complication occurring in the population. In the anastomotic safety model previously conducted at Maastricht University by Vogels et al. an anastomotic leakage rate of 23% (π) was encountered [2]. This results in a sample size of 12 animals, and taking a 10% drop-out in consideration, a group size of 14 animals per group.

Because of the drop-out in cohort 2 due to intestinal blood loss, we changed the sample size as described in the table below. We slightly adjusted the numbers in cohort 3 and 4 so that the expected intervention numbers based on the earlier sample size calculation in the working protocol (intended sample size of n = 12) was still statistically strong enough to draw a conclusion. As our research team has experience with sufficient anastomosis studies, we refer to these historical cohorts additional to our saline control group [3,4].

Group	Cohort 1	Cohort 2	Cohort 3	Cohort 4	Total
**Saline**	2	4	0	0	6
**Unloaded hydrogel**	2	4	6*	6*	18
**MMC-loaded hydrogel**	2	4	6*	6*	18

Experimental procedures

For surgical procedure, see manuscript and Supplementary 2 – Figure 1S. This was carried out in the rat operating room of the animal facility of Maastricht University under semi-sterile conditions.

Experimental animals

Forty-two Wistar rats (21 males/21 females) aged 10-12 weeks with a body weight of 400g – 500g (males) and 230g – 330g (females). Both male and female Wistar rats (RccHan:WIST) were purchased from Charles River Laboratories (Sulzfeld, Germany).

Housing and husbandry

An acclimatization period of one week was maintained prior to the start of the experiment. Rats were kept under standard conditions and were provided with food and water ad libitum. Rats were housed 2 animals per cage. The general health of rats was monitored several times per week for signs of

inflammation and animals were weighed once per week. During the experiment animals were weighed daily and scored for discomfort twice daily (every morning and every evening). In case of discomfort, additional pain treatment was administered by giving buprenorphine 0.05mg/kg s.c.
Discomfort was scored using a standard scheme (see below). Humane endpoints were defined according to Roughan & Flecknell (*Roughan, J. V. & Flecknell, P. A. Behavioural effects of laparotomy and analgesic effects of ketoprofen and carprofen in rats. Pain 90, 65–74 (2001).*

To monitor the risk of the development of AL (human end point (HEP)), welfare was scored daily. If an AL was expected, a veterinary was consulted at a certain welfare score (≥10) to discuss the care for the individual animal. Besides the risk of AL, we expected discomfort due to the intraperitoneal hydrogel itself. Based on the results of previous experiments, we expected a weight decrease of ~10% in the first week after administration as a result of the SMH degradation, but in this experiment this was expected to exceed to up to 15% as a result of the additional colon anastomosis. On days 0 and 1, animals received sufficient s.c. analgesia (buprenorphine). During the entire experiment (D0-7), paracetamol was provided in a second drinking bottle (next to normal drinking water). Intake of normal drinking water and paracetamol water was registered daily. Hydration status was monitored daily and if an animal was dehydrated, NaCl + 3% glucose as administered subcutaneously.

Summary of HEPs:

- Loss of > 15% weight loss within 3 days.
- Signs of peritonitis or severe intra-abdominal infection indicating AL → see welfare scoring sheet for more detailed information. Special attention was given to general signs of reduced discomfort (porphyria, piloerection, facial expression score), abdominal pain and/or peritonitis (distended abdomen, writhing, back arch, wincing, twitch, wet dog shake), and lack of stool/(bloody) diarrhea, and AL complications (colic signs with hypothermia or progressive increasing signs of colic not responding to analgesia or with ileus).
- In addition, body weight was recorded daily. In case of increasing discomfort, the veterinary was contacted at a discomfort score of 10 or higher to evaluate the individual risk of AL and decide if the animal needed additional analgesia or needed to be prematurely euthanized.

<u>Allocating animals to experimental groups</u>
The random allocation of the animals was done by a computer-based random order generator.

<u>Blinding</u>
During the allocation, the conduct of the experiment and the outcome assessment, the researcher team, the veterinarian, and the people working in the animal facility were blinded for the group allocation.

<u>Numbers analysed</u>
All analyses were performed according to an intention-to-treat analysis. However, since there were adverse events and deaths prior to follow-up, for AL score, adhesion score, bursting pressure and histological assessment these animals were not considered for the analysis.
For weight and survival, all animals who successfully underwent the operation were taken into account.

Animal Welfare Score Sheet													
**Cage number:**			**Animal ID:**										
**Date**													
**Day after surgery**	D	D	D	D	D	D	D	D	D	D	D	D	D
**Date and dose of additional analgesia administration**													
**Date and frequency of s.c. NaCl + 3% glucose administration**													
**Score normal is 0, score 1,2,3 for ↑ in severity (see description p.t.o.)**													
**General impression**													
**Awareness**													
**Posture**													
**Movement/activity**													
**Social behavior**													
**Coat condition**													
**Body condition score**													
**General physical examination and procedure specific indicators**													
**Signs of peritonitis/ colic:**<br>Combination of abdominal writhing, back arch, wincing, twitch, wet dog shake (assess frequency of behavior/ 15 min observation). Every time an indicator is observed, you score 1 point.<br>Example: 5x back arch and 3x twitch = combined score of 8 = 1 point on welfare scoring sheet													
**Body temperature**<br>Only necessary if the 'signs of peritonitis/colic' score ≥ 2													
**Facial expression score**													
**Porphyria score**													
**Hydration**													
**Feces**													
**Pica behavior**													
**Reaction when handled**													
**Total points**													
**INITIALS:**													

Description					
**General impression**					
	**0**	**1**	**2**	**3**	**HEP**
**Awareness**	Normal, alert	Slow, reduced		Apathy	Lifeless, coma
**Posture**	Normal	Hunched		Stagger (sudden loss of balance/gait),	Fall (falls over), circling Intention tremor
**Movement/activity**	Normal	Less/reduced activity	Markedly reduced activity	Significant mobility problems: persistent immobility<24h	Immobility >24h, reluctance to move, limping, seizure
**Social behavior**	Normal interaction	Reduced interaction with other animals/researcher	Significant reduced interaction with other animals/researcher or exploration		Isolated from cage mates, oblivion
**Coat condition**	Normal	Rough, Slightly unkempt	Slight pili-erection	Marked pili- erection	
**Body condition score** (BC1-BC 5) BC3 = well-conditioned	BC3	BC4		BC2	BC1 or BC5
**General physical examination and procedure specific indicators**					
	**0**	**1**	**2**	**3**	**HEP**
**Signs of peritonitis/colic combined score**	No signs or < 5x per 15 min observation	5-15x per 15 min. observation	> 15x per 15 min observation **Measure body temperature!**		
**Body temperature**	37,5 -38,5°C		>39 °C		> 40 °C or < 34,5 °C for 24 hours
**Facial expression**	Normal	Minor	Moderate	Severe	Severe>24h
**Porphyria score**	Absent	Present around eyes or nose	Present around eyes and nose	Profound discharge	
**Hydration**	Normal	Skin less elastic (delayed +/-)	Skin tents (delayed +)	Skin tents (delayed ++)	Skin tents(delayed +++), eyes sunken
**Feces**	Normal		Diarrhea	Blood in feces, absence of feces	Absence of any feces > 48h or absence with distended abdomen or > 48h bloody diarrhea
**Pica behavior**	Not present		Present		
**Reaction when handled**	Tense and nervous on handling	Markedly distressed on handling, e.g. shaking, vocalizing, aggressive			

**Actions to be taken**	**Total score**
Review frequency of monitoring	6
Consider supplementary fluids/analgesia	8
Review progress with bio-technician and consult veterinarian	10
Humane endpoint	12

[1] Viechtbauer, W., et al., *A simple formula for the calculation of sample size in pilot studies.* J Clin Epidemiol, 2015. **68**(11): p. 1375-9.
[2] Vogels, R.R., et al., *A new poly(1,3-trimethylene carbonate) film provides effective adhesion reduction after major abdominal surgery in a rat model.* Surgery, 2015. **157**(6): p. 1113-20.
[3] J W A M Bosmans and others, Functional mucous layer and healing of proximal colonic anastomoses in an experimental model, *British Journal of Surgery*, Volume 104, Issue 5, April 2017, Pages 619–630, https://doi.org/10.1002/bjs.10456
[4] Bosmans, J.W.A.M., Jongen, A.C.H.M., Boonen, B.T.C. *et al.* Comparison of three different application routes of butyrate to improve colonic anastomotic strength in rats. *Int J Colorectal Dis* **32**, 305–313 (2017). https://doi.org/10.1007/s00384-016-2718-z

S2: Supporting materials (results)

Table S1. Rat characteristics and outcomes

	**Saline**	**Unloaded hydrogel**	**MMC-loaded hydrogel**	***p* value**
**Number of rats** (n) – *(M/F)*	6 (3/3)	18 (9/9)	18 (9/9)	/
**Baseline weight M** (g) – *median (Q1 – Q3)*	408 (399 – 457)	416 (412 - 435)	430 (428 – 441)	0.269 [a]
**Baseline weight F** (g) – *median (Q1 – Q3)*	247 (233 - 248)	274 (246 – 298)	272 (261 – 282)	0.145 [a]
**Survival** (days) – *median (Q1 – Q3)*	7 (7 – 7)	7 (3 – 7)	3 (2 – 7)	**0.024** [a*]
**Completed the experiment** (n) – *(M/F)*	6 (3/3)	10 (6/4)	6 (1/5)	/

M, male; F, female; [a] Kruskal-Wallis test; *significant difference ($p = 0.009$) after pairwise comparison of NS and MMC-loaded hydrogel group with Mann-Whitney U test. No significant difference for saline compared to unloaded hydrogel ($p = 0.071$).

Table S2. Individual results of additional coagulation tests in four rats with (+) and one without (-) intestinal blood loss.

	**PT** (s)	**INR** (INR)	**aPTT** (s)	**Fibrinogen** (g/L)
**MMC-loaded hydrogel (+)**				
E 4.18 KL (M)	9.3	0.87	/	5.8
E 4.19 KL (M)	9.8	0.92	/	4.7
E 4.20 KL (F)	9.3	0.87	15	2.7
E 4.17 BL (M)	9.8	0.92	15	1.9
**MMC-loaded hydrogel (-)**				
E 4.22 BL (F)	9.2	0.86	/	2.1

/ = blood samples were too hemolytic
PT, prothrombin time; INR, International Normalized Ratio; aPTT, activated Partial Thromboplastin Time.

A previous study by García-Manzano et al. describes coagulation test values of normal male Wistar rats [1]. Although these rats were younger (200 – 300g), we compared our values to these previous described ones. The activated partial thromboplastin time (aPTT) ranged from 13.0 to 19.2s, which was similar to our results (15s) [1]. The prothrombin time (PT) ranged from 24.5 to 30.9s, which is higher than our observed PT times (9.2 – 9.8s). Lemini et al. also analyse coagulation levels in both adult male and female Wistar rats (200 - 300 g) [2]. They report PT times from 13.9 – 21.1s and 15.9 – 21.0 in male and female rats respectively. These values are closer to our study findings, which also counts for the aPTT and PT values reported by of Zhang et al. [3]. Fibrinogen ranged from 1.60 to 2.55 g/L in the rats from García-Manzano et al, and 2.00 – 3.01 g/L in male and 1.65 – 2.55 g/L in females. Three rats of our study cohort showed a value above this range. This can be explained by the fact that fibrinogen may be elevated in e.g. acute disease, inflammation, and in our case surgery as it is an acute phase protein. To summarize, we did not identify abnormal aPTT values, prolonged PT, or lower fibrogen levels than described in healthy rats, which means there are no coagulation problems on these levels. We were not able to find the international normalized ratio (INR) values for Wistar rats in the literature.

[1] García-Manzano A, González-Llaven J, Lemini C, Rubio-Póo C. Standardization of rat blood clotting tests with reagents used for humans. Proceedings of the Western Pharmacology Society. 2001;44:153-5.

[2] Lemini C, Jaimez R, Franco Y. Gender and inter-species influence on coagulation tests of rats and mice. Thrombosis Research. 2007;120(3):415-9.

[3] Zhang Y, Ying D, Liu H, Yu Z, Han L, Xie J, Xie Y. Serum pharmacokinetics and coagulation aberration induced by sodium dehydroacetate in male and female Wistar rats. Sci Rep. 2017 Apr 7;7:46210. doi: 10.1038/srep46210. PMID: 28387309; PMCID: PMC5384240.

Figure S1. Surgical procedure: creation of the anastomosis + closure of the fascia.

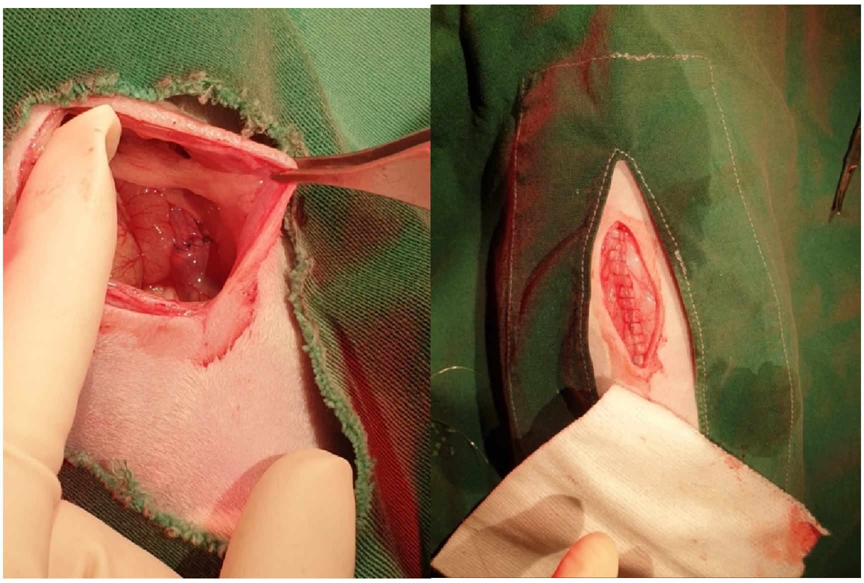

Figure S2. Bursting pressure measurement.

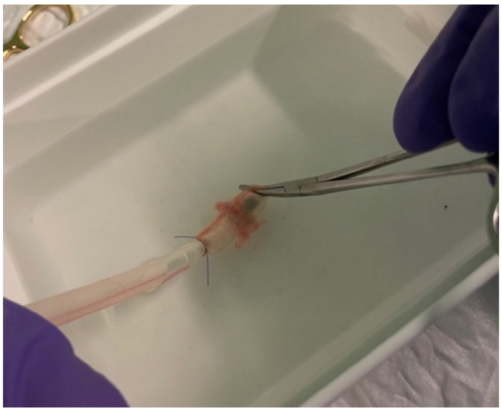

Figure S3. Survival proportions of the whole cohort.

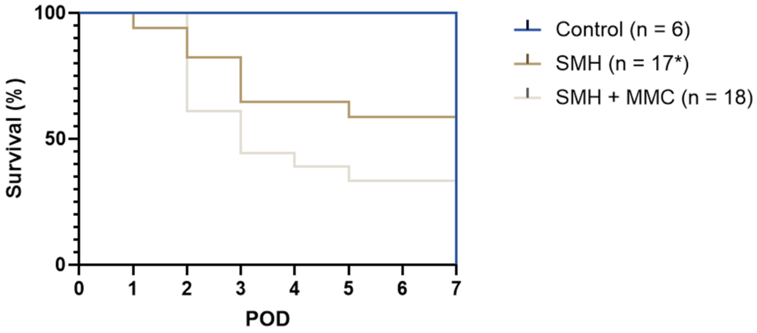

*One rat was excluded in the analysis due to a technical error during the operation.

Figure S4. Thrombocyte values of animals with (+) and without (-) blood loss. Medians are indicated and whiskers show the IQR.

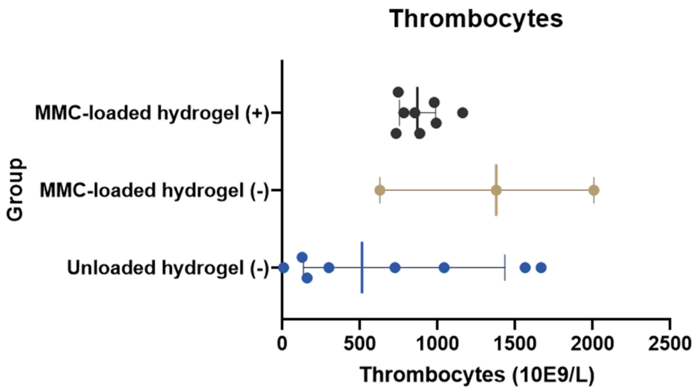

Figure S5. Median body weight changes in percentages compared to mean baseline weight in the 7 days of the experiment split for sex (whole cohort included, NS = 6, unloaded hydrogel = 17*, MMC-

loaded hydrogel = 18). Baseline weight is the weight at moment of the operation (100%). Whiskers show the IQR.

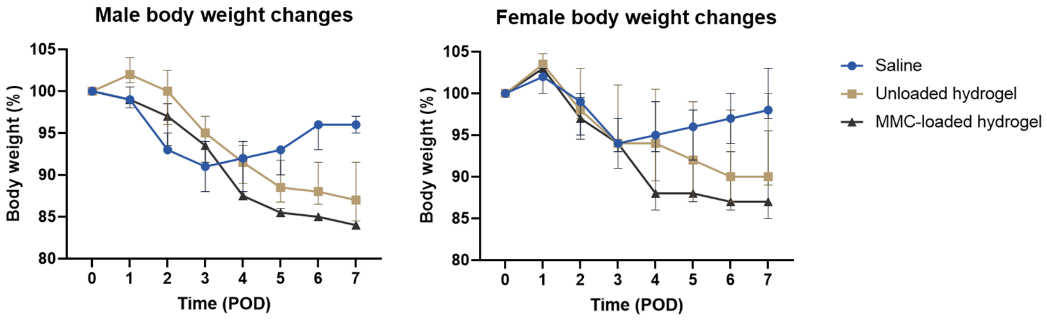

*One rat was excluded in the analysis due to a technical error during the operation.
